# Supplementary figures and images for: The Prognostic Role of Body Mass Index on Oncological Outcomes of Upper Tract Urothelial Carcinoma
Source: Cancers (Basel). 2023 Nov 10;15(22):5364. doi: 10.3390/cancers15225364 (PMC10670636; doi:10.3390/cancers15225364)

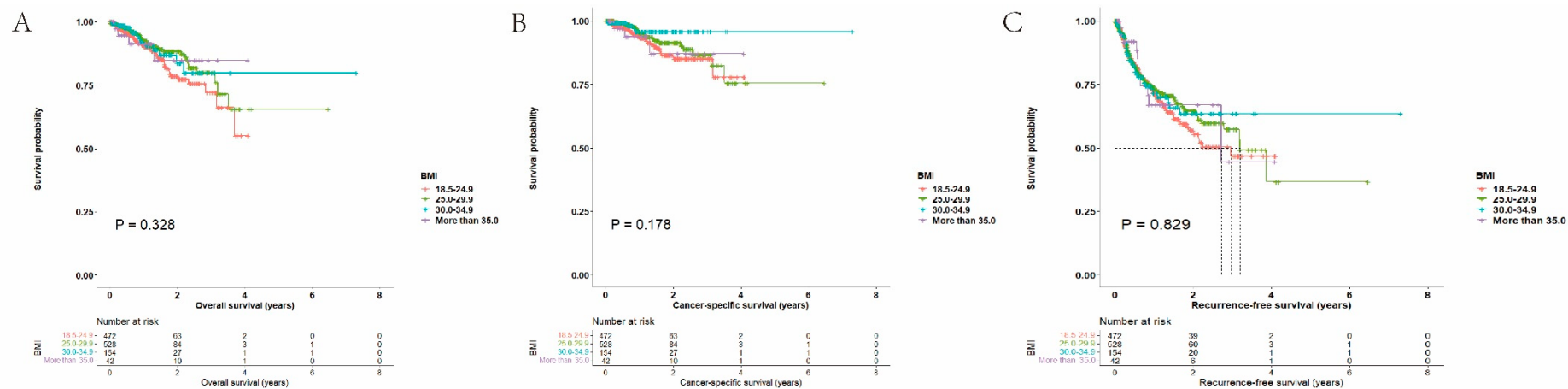

Supplementary Figure S1. Survival outcomes of subgroups. (A) OS, (B) CSS, (C) RFS.

Supplement: Supplementary file 1 [file cancers-15-05364-s001.zip › Supplementary Figure S1.pdf]
